# Is provider type associated with cancer screening and prevention: advanced practice registered nurses, physician assistants, and physicians

**DOI:** 10.1186/1471-2407-14-233

**Published:** 2014-03-31

**Authors:** Deanna Kepka, Alexandria Smith, Christopher Zeruto, K Robin Yabroff

**Affiliations:** 1 Division of Cancer Control and Population Sciences, National Cancer Institute, Bethesda, MD, USA; 2 College of Nursing, University of Utah, Salt Lake City, UT, USA; 3 Cancer Control and Population Sciences, Huntsman Cancer Institute, Salt Lake City, UT, USA; 4 American Legacy Foundation, Washington, DC, USA; 5 Information Management Services, Inc, Silver Spring, MD, USA

## Abstract

**Background:**

Physician recommendations for cancer screening and prevention are associated with patient compliance. However, time constraints may limit physicians’ ability to provide all recommended preventive services, especially with increasing demand from the Affordable Care Act in the United States. Team-based practice that includes advanced practice registered nurses and physician assistants (APRN/PA) may help meet this demand. This study investigates the relationship between an APRN/PA visit and receipt of guideline-consistent cancer screening and prevention recommendations.

**Methods:**

Data from the 2010 National Health Interview Survey were analyzed with multivariate logistic regression to assess provider type seen and receipt of guideline-consistent cancer screening and prevention recommendations (n = 26,716).

**Results:**

In adjusted analyses, women who saw a primary care physician (PCP) and an APRN/PA or a PCP without an APRN/PA in the past 12 months were more likely to be compliant with cervical and breast cancer screening guidelines than women who did not see a PCP or APRN/PA (all p < 0.0001 for provider type). Women and men who saw a PCP and an APRN/PA or a PCP without an APRN/PA were also more likely to receive guideline consistent colorectal cancer screening and advice to quit smoking and participate in physical activity than women and men who did not see a PCP or APRN/PA (all p < 0.01 for provider type).

**Conclusions:**

Seeing a PCP alone, or in conjunction with an APRN/PA is associated with patient receipt of guideline-consistent cancer prevention and screening recommendations. Integrating APRN/PA into primary care may assist with the delivery of cancer prevention and screening services. More intervention research efforts are needed to explore how APRN/PA will be best able to increase cancer screening, HPV vaccination, and receipt of behavioral counseling, especially during this era of healthcare reform.

## Background

With approval of the Affordable Care Act, millions of Americans who were without access to health insurance in 2010 will have affordable health insurance options by 2014 [1]. Furthermore, under the Affordable Care Act, Medicare and new health insurance plans and policies must cover selected evidence based preventive services without co-insurance, cost sharing, a copayment or contributions towards a deductible. These services include cervical, breast, and colorectal cancer screening. They also include human papillomavirus (HPV) vaccination for age eligible adults and tobacco use screening and cessation interventions [2,3].

Coverage of cancer-related screening and preventive services will increase the demand for the services of primary care physicians (PCP). However, the act of fulfilling all of the US Preventive Services Task Force recommendations at the appropriate frequency to an average size patient panel in the United States would consume an estimated 7.4 hours of an average US PCP’s workday, exclusive of treatment of illness and ongoing administrative activities [4]. In team-based practice settings, advanced practice registered nurses and physician assistants (APRN/PA) may help meet this demand for primary care. This approach has been suggested for improved organization of practice systems for chronic disease management since the 1980’s [5,6]. Additionally, a recent Institute of Medicine report (2011) called upon nurses to serve as full partners with physicians to help realize the goals of the Affordable Care Act [7]. APRNs are nurses with post-graduate education in nursing with an advanced scope of practice in nursing. The Affordable Care Act also provides for the Expansion of Physician Assistant (PA) Training Program as outlined by the US Department of Health and Human Services [8]. Physician assistants practice medicine alongside a physician supervisor with a similar scope of practice.

However, there are very few existing data sources to investigate these relationships between APRN/PA, other provider types, and receipt of care in relation to evidence-based guidelines at a national level. In this study, we used a theoretical framework to guide our evaluation of the relationship between type of medical provider seen in the past 12 months and receipt of cancer screening and prevention recommendations in a nationally representative sample. We hypothesized that individuals who have had a visit with an APRN/PA would be more likely to have received cancer screening and prevention recommendations than participants who have not seen a primary care provider or than participants who have not seen any healthcare provider.

## Methods

### Data and sample

Data from the publicly available 2010 National Health Interview Survey (NHIS) were used for this study. The NHIS is a nationally-representative cross-sectional survey of the civilian non-institutionalized population of the United States that employs a random, stratified, multi-stage cluster sampling design. It is conducted annually using computer-assisted in-person interviewing. The NHIS includes questions related to participant demographic characteristics, health, and healthcare use [9]. In 2010, the NHIS included a Cancer Control Supplement which contains sections on cancer screening, HPV vaccination, tobacco use, smoking cessation, and physical activity (n = 26,716) [9]. The Cancer Control Supplement had a response rate of 60.8% [9]. We used the sample weight assigned to each survey respondent, accounting for the probability of selection, as well as adjustment for nonresponse by sample strata.

### Theoretical framework

We used the Aday and Anderson theoretical framework to guide our selection of relevant variables and inform our analyses. The framework describes how healthcare utilization is influenced by health policies, characteristics of the available healthcare delivery system(s), and characteristics of the population at risk [10]. Aday and Anderson’s model can be applied empirically to assess how these factors impact access to health services. According to the model, the organizational structure of a healthcare system, such as the types of healthcare providers available, influence patients’ access to and receipt of recommended healthcare services [10]. Characteristics of a healthcare delivery system such as type of provider available and seen have been shown to influence treatment, self-care, and health outcomes in other studies [11,12]. Thus, the model was central to the selection and construct of the research question and outcomes of interest.

### Measures

The selection of the primary independent variable of interest is guided by the theoretical framework, which posits that the type of medical provider seen in the past 12 months has an effect on dependent variables, which include receipt of recommended cancer screening, HPV vaccination, and cancer prevention recommendations.

### Independent variables

#### Type of medical provider

The primary independent variable of interest was the type of medical provider, if any, seen in the past 12 months. Responses from multiple questions about provider type were combined to create the following mutually exclusive categories: 1. PCP (general practice, family medicine, internal medicine, and/or obstetrician/gynecologist) and an APRN/PA; 2. PCP and no APRN/PA; 3. Other provider (no PCP and no APRN/PA) such as a specialist, mental health professional, foot doctor, eye doctor, therapist, and/or chiropractor; and 4. No healthcare provider. Less than 300 out of a total of 15,171 women (prior to limiting by other factors such as age, classifiable provider type, and past cancer diagnoses) in the entire 2010 sample adult file indicated that they saw an APRN/PA without seeing either a general physician or an OB/GYN. Due to the very small number of participants who would have been eligible for our analyses and saw APRNs and PAs exclusively, we were unable to isolate distinct provider categories within those who saw an APRN/PA. PAs practice with an overseeing physician, and APRNs’ scope of independent practice and prescribing authority vary from state to state. As of 2013, only 16 states and the District of Columbia, granted APRNs independent diagnosing and prescribing authority [13]. This may have contributed to the small number of participants who saw an APRN/PA without seeing a PCP. In addition, participants were asked if they had seen a midwife (an APRN), a nurse practitioner (an APRN) or a physician assistant (PA) in the past 12 months in a single item on the NHIS. We were unable to distinguish between type of APRN seen in the past 12 months in these secondary data.

### Dependent variables

#### Recommended cancer screening, HPV vaccination, and cancer prevention recommendations

The US Preventive Services Task Force guidelines and the Advisory Committee on Immunization Practices recommendations were used to define cancer screening and prevention measures based on recommended time intervals and relevant age ranges [14-17]. Recommendations for smoking cessation among current smokers and recent former smokers were based on US Preventive Services Task Force guidelines [18]. Lastly, presence or absence of provider recommendations for physical activity in the past 12 months were also measured [19].

*Receipt of a Pap test* was investigated in the past three years among females aged 21 to 80 without a history of cervical cancer or receipt of a hysterectomy [15].

*HPV vaccine receipt* among females aged 18 – 30 years was explored [14] among females who confirmed whether or not they had ever received an HPV shot or vaccine. Females who stated that their doctor refused when asked were classified as not receiving the HPV shot.

*Receipt of a mammogram* in the past two years among females aged 40 to 80 years without a history of breast cancer was looked into [16].

*Colorectal cancer screening* was measured as receipt of a colonoscopy within the past 10 years, a sigmoidoscopy within the past 5 years, and/or a home fecal occult blood test (FOBT) within the past year among females and males separately, aged 50 to 80 years without a history of colorectal cancer [17].

*Smoking cessation recommendations* within the past 12 months were explored among current or recent former smokers aged 18 and older for females and males separately [18].

*Physical activity recommendations* were measured as a receipt of advice by a health care provider to begin or continue any type of exercise or physical activity within the past 12 months, and were investigated among females and males separately, aged 18 and older [19].

### Patient characteristics

As described in our theoretical model, patient demographic characteristics, health status, and type of health insurance, if any, are associated with healthcare utilization. We selected individual characteristics that have been shown elsewhere to be associated with cancer screening and prevention recommendations, including age, level of education, health insurance status, and health status [20]. We measured health status as the number of health conditions identified by a health provider. Additionally, because interaction with the healthcare system results in more opportunities for recommendations for cancer screening and prevention, we also adjust for number of provider visits in the past 12 months.

### Data analysis

Sample characteristics were stratified by provider type (PCP and an APRN/PA; PCP and no APRN/PA; other healthcare provider; no provider). The associations between participant characteristics and use of a healthcare provider in the past 12 months by type of provider seen were assessed using the Wald F-test. Adjusted proportions (predicted marginals) were calculated using logistic regression models that were stratified by gender to investigate the relationship between type of provider seen in the past 12 months and receipt of cancer screening or prevention recommendations. Based on our theoretical framework, our multivariate models adjusted for age, level of education, insurance status, health status and number of provider visits in the past 12 months. Sensitivity analyses were conducted to evaluate the impact of different lower and upper age limits in models of cancer screening. All estimates accounted for the stratification and clustering of data within the complex survey design of NHIS using multiple stage survey functions. SAS and SUDAAN statistical software were used to conduct all statistical analyses. Analyses of public use data are considered exempt by the Institutional Review Board (IRB) of the National Cancer Institute.

## Results

### Participant characteristics

Table 1 presents the participant characteristics and use of provider(s) by type. 19.2% of participants saw a PCP and an APRN/PA and 55.3% saw a PCP and no APRN/PA, 7.5% saw a different health provider and no APRN/PA, and 18.0% did not see any healthcare provider in the past 12 months. All demographic characteristics (gender, age, education, income, and health insurance type) were associated with type of provider seen in the past 12 months (p < 0.0001).

**Table 1:** **Participant characteristics and use of healthcare provider in the past 12 months by type of provider seen, National Health Interview Survey 2010 (n = 26,716)**^**$**^

	**Have seen or talked with any provider**		**Seen or talked with a primary care physician and no APRN/PA**^**a**^		**Seen or talked with a primary care physician and an APRN/PA**^**b**^		**Seen or talked with other healthcare provider**^**c **^**and no APRN/PA**^**b**^		**Did not see any healthcare provider**	
	**N**	**(%)**	**N**	**(%)**	**N**	**(%)**	**N**	**(%)**	**N**	**(%)**
**Total**	21,752	(82.0)	14,944	(55.3)	4,891	(19.2)	1,917	(7.5)	4964	(18.0)
**Gender**										
Male	8,657	(44.2)	5,790	(43.3)	1,745	(39.2)	1,122	(63.6)	3,125	(67.2)
Female	13,095	(55.8)	9,154	(56.7)	3,146	(60.8)	795	(36.4)	1,839	(32.8)
**Age, y**										
18-20	726	(4.8)	496	(5.0)	141	(4.0)	89	(5.7)	296	(8.0)
21-30	3,393	(16.3)	2,159	(14.9)	842	(17.9)	392	(21.8)	1,304	(28.7)
31-49	6,979	(32.9)	4,678	(32.0)	1,601	(33.5)	700	(37.7)	2,107	(40.5)
50-64	5,655	(26.7)	3,884	(26.9)	1,333	(27.9)	438	(22.3)	908	(17.5)
65-80	3,731	(14.9)	2,743	(16.1)	753	(13.4)	235	(10.1)	285	(4.4)
81+	1,268	(4.4)	984	(5.0)	221	(3.4)	63	(2.4)	64	(1.0)
**Education (missing N = 114)**										
Less than high school	3,286	(12.6)	2,524	(14.0)	479	(8.5)	283	(12.7)	1,280	(22.6)
High school graduate or GED	5,519	(25.6)	3,856	(25.9)	1,146	(23.8)	517	(27.6)	1,531	(32.7)
Some college or AA degree, less than 4 year degree^d^	6,691	(31.4)	4,406	(30.2)	1,725	(35.3)	560	(30.4)	1,239	(26.4)
College graduate BA/BS, or higher^e^	6,166	(30.4)	4,097	(29.9)	1,519	(32.4)	550	(29.3)	890	(18.3)
**Health insurance (missing N = 79)**										
Uninsured	2,635	(11.6)	1,700	(10.7)	498	(10.0)	437	(22.0)	2,381	(46.9)
Any private/military	14,417	(70.8)	9,823	(70.5)	3,432	(74.1)	1162	(65.5)	1,935	(42.9)
Public only	4,640	(17.3)	3,377	(18.4)	953	(15.7)	310	(12.5)	629	(10.2)
**Number of healthcare provider visits (missing N = 116)**										
0-1	5,022	(23.4)	3,383	(22.9)	518	(10.7)	1121	(59.4)	4,456	(90.7)
2-3	6,445	(30.2)	4,891	(33.6)	1,185	(25.0)	369	(19.1)	336	(6.5)
4+	10,179	(46.3)	6,594	(43.5)	3,163	(64.3)	422	(21.5)	162	(2.8)
**Number of health conditions**										
0	7,517	(35.7)	5,262	(36.3)	1,342	(29.2)	913	(47.9)	3,319	(67.3)
1	5,331	(25.7)	3,691	(25.9)	1,148	(24.7)	492	(27.0)	1,063	(21.5)
2	3,579	(16.2)	2,463	(16.1)	855	(17.5)	261	(13.2)	338	(6.5)
3+	5,325	(22.4)	3,528	(21.7)	1,546	(28.6)	251	(11.9)	244	(4.6)

Note: Chi Square Test used to test for association between type of provider seen and participant characteristic. Percentages are weighted estimates.

^$^The associations between participant characteristics and use of a healthcare provider in the past 12 months by type of provider seen were assessed using the Wald F-test. Each association was statistically significant at p < .01.

^a^Primary care physician includes physicians in general practice, family medicine, internal medicine, and obstetrics/gynecology. APRN indicates Advanced Practice Registered Nurse which includes certified nurse midwives (CNM) and nurse practitioners (NP); PA indicates physician assistant.

^b^APRN indicates Advanced Practice Registered Nurse which includes certified nurse midwives (CNM) and nurse practitioners (NP); PA indicates physician assistant.

^c^Other healthcare providers include mental health professionals such as a psychiatrists, psychologists, psychiatric nurses, or clinical social workers. It also includes foot doctors, chiropractors, physical therapists, speech therapists, respiratory therapists, audiologists, and/or occupational therapists.

^d^AA indicates Associate in Arts.

^e^BA indicates Bachelor of Arts; BS indicates Bachelor of Science.

### Cancer screening and HPV vaccination

Table 2 presents receipt of a Pap test, mammogram, and the HPV vaccine by provider type among women. Receipt of cancer screening (cervical and breast) was associated with type of provider seen type (PCP and an APRN/PA; PCP and no APRN/PA; other healthcare provider) in the past 12 months in adjusted analyses (p < 0.0001). Age-eligible women were more likely to receive Pap screening if they saw a PCP and an APRN/PA (90.0%) or PCP and no APRN/PA (85.6%) compared to those who saw another type of healthcare provider (67.7%) or no healthcare provider (66.8%). Women were also more likely to receive a mammogram if they saw a PCP and an APRN/PA (74.0%) or PCP and no APRN/PA (74.6%) compared to those who saw another type of healthcare provider (53.5%) or no healthcare provider (49.3%).

**Table 2:** Adjusted proportions of receipt of a Pap test, HPV vaccination, and mammogram by provider type, 2010 National Health Interview Survey

	**Received a Pap test within past 3 years, females age 21–80 years**^**a**^		**Ever Received HPV shot or vaccine, females age 18–30 years**		**Received a mammogram within past 2 years, females age 40–80 years**^**b**^	
	**Unadjusted (N = 9,997)**	**Adjusted (N = 9,933)**	**Unadjusted (N = 2,891)**	**Adjusted (N = 2,868)**	**Unadjusted (N = 7,601)**	**Adjusted (N = 7,543)**
Wald F test p-value	p < .0001	p < .0001	p < .001	p = .30	p < .0001	p < .0001
Seen or talked with in past 12 months:	% (95% CI)	% (95% CI)	% (95% CI)	% (95% CI)	% (95% CI)	% (95% CI)
Primary care physician^d^ & APRN/PA^c^	92.4 (91.0-93.5)	90.0 (88.2–91.6)	19.9 (16.1–24.3)	18.4 (14.9–22.4)	78.2 (75.8-80.4)	74.0 (71.5-76.4)
Primary care physician^d^ and no APRN/PA^c^	86.3 (85.1-87.5)	85.6 (84.4–86.8)	19.9 (17.6–22.6)	19.3 (16.9-21.9)	76.2 (74.6-77.7)	74.6 (73.0-76.2)
Other healthcare provider^e^ and no APRN/PA^c^	61.2 (56.1-66.1)	67.7 (63.1–72.1)	12.1 (7.1-19.8)	13.7 (8.0-22.4)	47.5 (41.4-53.6)	53.5 (47.4-59.5)
No healthcare provider	53.5 (50.3-56.7)	66.68 (63.3–70.1)	9.2 (6.1–13.6)	12.8 (8.1-19.6)	30.9 (27.2-34.8)	49.3 (44.5-54.2)

Note: Adjusted proportions or predicted marginals were calculated using multivariate logistic regression models that adjust for age, level of education, insurance status, number of chronic conditions, and number of healthcare provider visits in the past year.

^a^Excludes women with a past diagnosis of cervical cancer and women who have undergone a hysterectomy.

^b^Excludes women with a past diagnosis of breast cancer.

^c^APRN indicates Advanced Practice Registered Nurse which includes certified nurse midwives (CNM) and nurse practitioners (NP); PA indicates physician assistant.

^d^Primary care physician includes physicians in general practice, family medicine, internal medicine, and obstetrics/gynecology.

^e^Other healthcare providers include mental health professionals such as a psychiatrists, psychologists, psychiatric nurses, or clinical social workers. It also includes foot doctors, chiropractors, physical therapists, speech therapists, respiratory therapists, audiologists, and/or occupational therapists.

Table 3 presents compliance with colorectal cancer screening guidelines. In adjusted analyses, women were more likely to have received any of the recommended colorectal cancer screening tests at the appropriate interval if they saw a PCP and an APRN/PA (64.2%) or PCP and no APRN/PA (61.9%) compared to those who saw another type of healthcare provider (42.5%) or no healthcare provider (43.8%). Men were also more likely to receive recommended colorectal cancer screening tests if they saw a PCP and an APRN/PA (63.9%) or PCP and no APRN/PA (62.8%) compared to those who saw another type of healthcare provider (54.2%) or no healthcare provider (44.7%).

**Table 3:** **Adjusted proportions of compliance with colorectal cancer screening recommendations by provider type, 2010 National Health Interview Survey**^**a**^

	**Females age 50-80**		**Males age 50-80**	
	**Unadjusted (N = 5,449)**	**Adjusted (N = 5,397)**	**Unadjusted (N = 4,193)**	**Adjusted (N = 4,162)**
Wald F test p-value	p < .0001	p < .0001	p < .0001	p < .0001
Seen or talked with in past 12 months:	% (95% CI)	% (95% CI)	% (95% CI)	% (95% CI)
Primary care physician^c^ & APRN/PA^b^	69.2 (65.9–72.3)	64.2 (60.8-67.4)	71.3 (67.4-75.0)	63.9 (60.0-67.6)
Primary care physician^c^ and no APRN/PA^b^	63.3 (61.3–65.3)	61.9 (59.9-63.9)	65.8 (63.6-67.9)	62.8 (60.6-64.9)
Other healthcare provider^d^ and no APRN/PA^b^	36.1 (29.8-43.0)	42.5 (35.7-49.7)	48.4 (42.0-54.7)	54.2 (48.3-59.9)
No healthcare provider	24.9 (20.3-30.2)	43.8 (37.1-50.8)	22.9 (19.1-27.3)	44.7 (39.4-50.1)

Note: Adjusted proportions or predicted marginals were calculated using multivariate logistic regression models that adjust for age, level of education, insurance status, number of chronic conditions, and number of healthcare provider visits in the past year and participants with a past diagnosis of colon and/or rectal cancer were excluded.

^a^Compliance with colorectal screening guidelines was defined as a colonoscopy in the past 10 years, a sigmoidoscopy in the past 5 years, and/or a home FOBT in the past year according to the US Preventive Services Task Force Guidelines (2008).

^b^APRN indicates Advanced Practice Registered Nurse which includes certified nurse midwives (CNM) and nurse practitioners (NP); PA indicates physician assistant.

^c^Primary care physician includes physicians in general practice, family medicine, internal medicine, and obstetrics/gynecology.

^d^Other healthcare providers include mental health professionals such as a psychiatrists, psychologists, psychiatric nurses, or clinical social workers. It also includes foot doctors, chiropractors, physical therapists, speech therapists, respiratory therapists, audiologists, and/or occupational therapists.

Sensitivity analyses including different lower and upper age limits for cancer screening analyses (e.g., receipt of a Pap test among women age 21–80 vs. 21–69 years) had little impact on findings. Notably, participants who had seen a primary care provider (PCP and an APRN/PA, or PCP and no APRN/PA) in each model were more likely to meet cancer screening guidelines.

### Risk reduction recommendations

Receipt of smoking cessation and physical activity recommendations were also associated with type of provider seen in the past 12 months in adjusted analyses (all p < .01; Table 4). Women were more likely to receive tobacco cessation recommendations if they saw a PCP and an APRN/PA (55.6%) or PCP and no APRN/PA (55.7%) than those who saw other types of healthcare providers (36.1%). Men were more likely to receive tobacco cessation recommendations if they saw a PCP and an APRN/PA (57.7%) or PCP and no APRN/PA (53.2%) than those who saw other types of healthcare providers (36.0%). Furthermore, women and men were more likely to report receipt of physical activity recommendations if they saw a PCP and an APRN/PA (38.0% and 35.8% respectively) or PCP and no APRN/PA (35.9% and 32.9%, respectively) than those who saw other types of healthcare providers (28.4% and 26.4%, respectively).

**Table 4:** Adjusted proportions of receipt of behavioral counseling in the past 12 months from provider by provider type, 2010 National Health Interview Survey, adults aged ≥18

	**Received advice to quit smoking from a doctor, dentist, or other health professional**^**a**^				**Received a recommendation to begin or continue any type of physical activity from a doctor or other health professional**^**b**^			
	**Females**		**Males**		**Females**		**Males**	
	**Unadjusted (N = 2,196)**	**Adjusted (N = 2,153)**	**Unadjusted (N = 1,705)**	**Adjusted (N = 1,678)**	**Unadjusted (N = 11,752)**	**Adjusted (N = 10,870)**	**Unadjusted (N = 7,544)**	**Adjusted (N = 7,126)**
Wald F test p-value	p < .001	p < .01	p < .0001	p < .001	p < .0001	p < .001	p < .0001	p < .001
Seen or talked with in past 12 months:	% (95% CI)	% (95% CI)	% (95% CI)	% (95% CI)	% (95% CI)	% (95% CI)	% (95% CI)	% (95% CI)
Primary care physician^d^ & APRN/PA^c^	57.6 (53.0-62.1)	55.6 (51.0-60.2)	62.0 (56.3-67.4)	57.7 (51.8-63.5)	40.2 (38.3-42.2)	38.0 (36.1-40.0)	40.1 (37.2-43.1)	35.8 (33.0-38.8)
Primary care physician^d^ and no APRN/PA^c^	54.8 (51.9-57.7)	55.7 (52.8-58.7)	52.5 (48.9-56.0)	53.2 (49.6-56.8)	34.7 (33.4-36.1)	35.9 (34.5-37.3)	31.8 (30.3-33.4)	32.9 (31.4-34.5)
Other healthcare provider^e^	33.7 (24.1-44.9)	36.1 (26.0-47.7)	29.3 (22.4-37.4)	36.0 (27.7-45.2)	26.1 (21.7-31.1)	28.4 (23.6-33.8)	22.7 (19.2-26.7)	26.4 (22.4-30.9)

Note: Adjusted proportions or predicted marginals were calculated using multivariate logistic regression models that adjust for age, level of education, insurance status, number of chronic conditions, and number of healthcare provider visits in the past year.

^a^Sample adults who have seen a doctor or other health professional in the past 12 months and are current smokers or former smokers who have quit in the past 12 months.

^b^Sample adults who have seen a doctor or other health professional in the past 12 months.

^c^APRN indicates Advanced Practice Registered Nurse which includes certified nurse midwives (CNM) and nurse practitioners (NP); PA indicates physician assistant.

^d^Primary care physician includes physicians in general practice, family medicine, internal medicine, and obstetrics/gynecology.

^e^Other healthcare providers include mental health professionals such as a psychiatrists, psychologists, psychiatric nurses, or clinical social workers. It also includes foot doctors, chiropractors, physical therapists, speech therapists, respiratory therapists, audiologists, and/or occupational therapists.

## Discussion

For more than two decades, team-based healthcare has been known to play a key role in improving primary care [5,6,21,22], yet this is one of the first studies to use nationally-representative data to investigate the relationship between provider type, including PCP, APRN and/or PA, and other providers and receipt of cancer screening and cancer risk reduction recommendations. Overall, type of provider seen in the past 12 months, if any, was associated with receipt of guideline-consistent cancer risk reduction recommendations and cancer screening. These findings suggest that seeing a PCP alone, or in addition to an APRN/PA is associated with greater receipt of a wide range of cancer prevention services and recommendations compared to not seeing any provider or another provider type even when controlling for number of provider visits in the past year. The importance of a visit with a primary care provider was underscored by the findings of this study. These findings are supported by previous literature that suggests the availability of primary care physicians is one of the most influential factors related to self-rated health, public health, and population health outcomes [23-25].

Our study adds to the limited literature assessing the effects of an APRN/PA visit on cancer risk factor reduction and screening recommendations. Only a small number of studies have evaluated cancer screening and risk reduction recommendations by provider type or by APRN/PA. These studies generally report high levels of Pap test, mammography or colorectal cancer screening ordered or performed by nurse practitioners (NP) [26-30] and in some situations NP had better performance than their physician counterparts [30]. Other studies have shown that patients interacting with NP, certified nurse midwives (CNM), or PA in both primary care facilities and hospitals are likely to receive smoking cessation counseling [26,27,31-35]. In addition, a few studies have shown that NP are more likely to counsel on diet and physical activity than their physician counterparts [27,31,32] while some noted that APRN/PA provide counseling on physical activity to less than a quarter of their patients [27,32]. These studies generally had small samples and rarely had comparison groups or evaluated multiple cancer control interventions in the same populations. Further, none of these studies reported cancer screening or risk reduction recommendations in relation to evidence-based guidelines.

More work is needed to identify the optimal structure of primary care provider teams for improving delivery of cancer screening and prevention recommendations. Findings will aid in the further development and design of US healthcare systems to meet the expected demands for primary care as a result of the Affordable Care Act.

This study’s findings highlight the importance of identifying provider type when evaluating delivery of primary care services. In several instances, associations between receipt of cancer screening and seeing a non-primary care provider in the past 12 months was similar to not seeing any provider at all. More work is needed to understand the roles of PCP and APRN/PA separately and as a part of primary care teams as related to increasing compliance with cancer screening and prevention recommendations. A combination of in-depth qualitative interviews and quantitative surveys with patients and providers within and between healthcare systems could inform the development and evaluation of interventions integrating APRN/PA in primary care teams. Because vulnerable populations in the United States, including the uninsured, low-income, and minorities, are less likely to be compliant with cancer screening and prevention recommendations [36] they may be a particularly important target for intervention activities, particularly in safety net clinics.

Unlike breast and cervical cancer screening guidelines, which are based on intervals for mammography and Pap testing, there are a number of test options with different recommended intervals for meeting colorectal cancer screening guidelines (i.e., FOBT, sigmoidoscopy, and colonoscopy). Thus, patients may need more provider time to guide their decision-making processes. Further, on a national level, compliance with colorectal cancer screening guidelines is low at 59%, compared to compliance with breast (72%) and cervical cancer (83%) screening guidelines among age eligible adults [37]. In addition to having more room for improvement, colorectal cancer screening assessments are less vulnerable to a ceiling effect. Thus, learning how to optimally integrate APRN/PA into primary care teams may lead to greater improvements in colorectal cancer screening rates than would be feasible for breast and cervical cancer screening rates. Furthermore, there is also large room for improvement to increase advice to quit smoking and to offer physical activity recommendations where integration of APRN/PA in primary care may help improve these low rates (less than 60% of participants received advice to quit smoking and less than 40% of participants received a recommendation to begin or continue physical activity).

Few studies have investigated the role of APRN/PA in HPV vaccination, although NP working with adolescents are more likely to recommend STI vaccines [38] and CNM are active in the assessment of the need for vaccinations among their patients [26]. Future work in this area should investigate the relationship between a visit with an APRN/PA and receipt of the HPV vaccine within pediatrics clinics to better capture the patient populations (girls and boys aged 11 and 12 years) who would receive the most benefit from the vaccine.

This study exhibits several limitations. First, the data do not allow for a separate analysis of the services specifically provided by APRN/PA in relation to those that are provided by PCP. Secondly, it is unknown if participants saw an APRN/PA at the same visit and in the same practice as their PCP. Athough it is impossible to assess the impact of a provider team on receipt of cancer screening and prevention recommendations, most states do require that APRN/PA practice with a PCP. Furthermore, the dates of cancer screening test, HPV vaccination, and provider recommendations for cancer prevention and specific date of each provider visit are not available. For colorectal cancer screening, this study assessed receipt of screening tests within a recommended time interval for type of test received (i.e., home FOBT in the past year, sigmoidoscopy within 5 years, colonoscopy within 10 years) yet participants were asked about visits with primary care providers and other healthcare providers in the past 12 months. However, this analysis is likely to underestimate receipt of colorectal cancer screening received five or more years ago. Lastly, these data were gathered from self-report survey items. Participant recall of the specific type of medical provider(s) seen in the past 12 months and participant accuracy in reporting of the timing of type of medical recommendation, procedure, and/or service received is an additional limitation. Self-report of cancer screening may overestimate receipt of screening [39-41], although some studies demonstrate reliable agreement of self-report screening [42,43] as compared to physician reports and medical records.

## Conclusions

Opportunities exist to increase the role of APRN/PA in cancer prevention and control to respond to a growing demand for affordable preventive services in primary care settings in the United States. APRN/PA may be a strong leverage point to heighten cancer-prevention among vulnerable populations. More intervention research efforts are needed to explore how APRN/PA will be best able to increase cancer screening, HPV vaccination, and receipt of behavioral counseling, especially during this era of healthcare reform, as both individual practitioners and as members of healthcare delivery teams. Lastly, increasing overall access to primary care providers will likely improve the delivery of cancer-prevention services in the United States.

## Competing interests

We do not have any competing interests to Report.

## Authors’ contributions

DK conceived of the research question, led the data analysis activities and wrote the manuscript. LS completed the literature review, and wrote and edited the manuscript. CZ completed the data analysis activities and reviewed the manuscript. RY helped DK oversee the study, wrote sections of the manuscript, and reviewed, and edited the manuscript. All authors read and approved the final manuscript.

## Pre-publication history

The pre-publication history for this paper can be accessed here:

http://www.biomedcentral.com/1471-2407/14/233/prepub
